# Combination of Three Herbal Components (ISL, Que, Meth) Suppresses Uveal Melanoma Growth via Gαq/MEK/YAP Axis Modulation and Apoptosis

**DOI:** 10.3390/biomedicines14071596

**Published:** 2026-07-16

**Authors:** Xiqianru Zhang, Rouqing Wu, Chengdan Yan, Ruifeng Wang, Yuemei Zhang

**Affiliations:** 1 The First School of Clinical Medicine, Lanzhou University, Lanzhou 730000, China; zhangxqr2024@lzu.edu.cn (X.Z.); 17262869202@163.com (R.W.); yanchd2025@lzu.edu.cn (C.Y.); 2 Key Laboratory of Dunhuang Medical and Transformation, Ministry of Education of The People’s Republic of China, Gansu University of Chinese Medicine, Lanzhou 730000, China; wangruifeng0526@163.com; 3 Department of Ophthalmology, The First Hospital of Lanzhou University, Lanzhou 730000, China

**Keywords:** uveal melanoma (UM), Isoliquiritigenin (ISL), Methylnissolin (Meth), Quercetin (Que), synergistic therapy, apoptosis, Gαq/MEK/YAP axis

## Abstract

**Background:** Uveal melanoma (UM) represents the most prevalent primary intraocular malignancy in adults, yet patients harboring *GNAQ/GNA11* mutations face particularly poor prognoses with median survival of merely 6–12 months following metastasis. Multi-targeted combination therapy offers a promising strategy to circumvent drug resistance. The present study investigated the synergistic anti-tumor efficacy and mechanistic basis of Isoliquiritigenin (ISL), Quercetin (Que) and Methylnissolin (Meth), three bioactive constituents from *Astragalus membranaceus* (*Huangqi*, a widely used traditional Chinese medicinal herb) against UM. **Methods:** Molecular docking and 100 ns molecular dynamics simulations assessed binding stability between the compounds and their respective targets (Gαq, MEK and YAP). Synergistic interactions were quantified using the Zero Interaction Potency (ZIP) model, a reference synergy model that compares observed combination effects to predicted non-interaction baselines across full dose–response matrices, based on CCK-8 assays. Cell cycle distribution, apoptosis and mitochondrial membrane potential were analyzed by flow cytometry. Western blotting detected target proteins and apoptotic markers. A male BALB/c nude mouse xenograft model validated therapeutic efficacy and systemic safety. **Results:** Molecular docking revealed binding energies <−7.0 kcal·mol^−1^ for all three drug–target pairs, with molecular dynamics trajectories confirming stable complex conformations (RMSD < 3 Å). In vitro, the ISL-Que-Meth (IQM) combination exhibited strong synergism (ZIP scores > 10), significantly increasing apoptotic rates, collapsing mitochondrial membrane potential and upregulating cleaved-caspase 9 expression compared with monotherapy, and a modest G2/M phase accumulation was also observed, although the magnitude was limited relative to apoptotic induction. In vivo, the triple combination achieved approximately 50% reduction in tumor growth compared with the control group, with effects comparable to or exceeding those of the clinical reference agent Trametinib, and reduced Ki67 proliferation indices while elevating cleaved-caspase 9 levels, without eliciting hepatorenal toxicity. While these data demonstrate therapeutic efficacy, they do not establish in vivo synergy, as single-agent and dual-combination arms were not included in the xenograft design. **Conclusions:** These findings demonstrate that IQM synergistically suppresses UM growth in association with coordinated modulation of Gαq/MEK/YAP axis components and caspase 9-dependent apoptosis via the intrinsic mitochondrial pathway, providing preclinical evidence for natural product-based multi-targeted therapy against UM.

## 1. Introduction

Uveal melanoma (UM) is the most prevalent primary intraocular malignancy in adults. Despite effective local control through surgery or radiotherapy, approximately 50% of patients eventually develop distant metastases, predominantly to the liver, with a median survival of merely 6–12 months [1,2]. Genetic investigations have identified activating mutations in *GNAQ* or *GNA11* in roughly 85% of UM cases [3]. These oncogenic mutations drive constitutive activation of Gαq protein, subsequently hyperactivating downstream Hippo/YAP and MAPK/ERK signaling cascades to promote uncontrolled proliferation, survival and metastasis. However, Gαq signaling can also be activated through alternative mechanisms in tumors lacking these mutations [4,5,6]. Consequently, targeted intervention against Gαq signaling and its downstream effectors has emerged as a critical therapeutic imperative across UM subtypes.

Current systemic therapies for metastatic UM primarily comprise MEK inhibitors (e.g., Trametinib) and immune checkpoint inhibitors [7,8,9]. However, single-target agents frequently encounter resistance arising from pathway feedback activation or compensatory bypass mechanisms. Furthermore, their clinical utility remains constrained by substantial toxicities [10,11]. Therefore, simultaneous inhibition of multiple nodes within the oncogenic signaling network represents a rational approach to overcome resistance and improve therapeutic outcomes [12,13,14].

Modern pharmacological investigations have revealed that *Astragalus membranaceus* harbors diverse bioactive constituents capable of exerting anti-tumor effects through multi-target mechanisms [15,16,17,18,19,20]. Our preliminary experiments identified Isoliquiritigenin (ISL), Quercetin (Que) and Methylnissolin (Meth) as active components targeting Gαq, MEK and YAP, respectively. These three agents theoretically target the Gαq/MEK/YAP axis, suggesting potential for synergistic interaction.

To the best of our knowledge, however, the combinatorial anti-tumor efficacy of this triple regimen against UM and its underlying molecular mechanisms remain unexplored. The present study employed an integrated strategy to systematically investigate the cooperative inhibitory effects of ISL-Que-Meth (IQM) against UM, with specific emphasis on elucidating the activation of caspase 9-dependent apoptosis via the intrinsic mitochondrial pathway as the primary anti-tumor mechanism. To evaluate the generalizability of this multi-targeted strategy, we selected two UM cell lines with distinct genetic backgrounds: 92.1 (*GNAQ* Q209L mutant) and C918 (*GNAQ/GNA11* wild-type). The inclusion of a wild-type cell line allowed us to determine whether the therapeutic efficacy is dependent on oncogenic mutation or rather reflects fundamental dependency on Gαq/MEK/YAP axis activity in UM.

## 2. Materials and Methods

### 2.1. Cell Culture and Reagents

Human UM cell lines 92.1 (*GNAQ* mutant) (IMMOCELL, Xiamen, China, cat. no. IM-H649) and C918 (*GNAQ/11* wild-type) (Procell, Bethel, CT, USA, cat. no. CL-0264), and human retinal pigment epithelial cell line ARPE were cultured in RPMI-1640 medium (Gibco, New York, NY, USA, cat. no. AF29520450) supplemented with 10% fetal bovine serum (FBS, Grand Island, NY, USA; Gibco, cat. no. 1919555) and 1% penicillin/streptomycin (BI, Beit Haemek, Israel; cat. no. C3423-0100) at 37 °C in a humidified atmosphere containing 5% CO_2_. ISL (TargetMol, Shanghai, China, cat. no. T0725; purity ≥ 97.61%), Que (TargetMol, cat. no. T2174; purity ≥ 97.1%), Meth (TargetMol, cat. no. TN2354; purity ≥ 99.78%) and Trametinib (TargetMol, cat. no. T2125; purity ≥ 99.96%) were dissolved in dimethyl sulfoxide (DMSO) to prepare 10 mmol/L stock solutions and stored at −20 °C. The final DMSO concentration in culture medium did not exceed 0.1%. (All cell lines were authenticated by STR profiling. The STR report for C918 is provided in Supplementary Data S1; the STR profile of 92.1 (IMMOCELL, IM-H649) is publicly documented in Cellosaurus (CVCL_8607) and provided as Supplementary Data S2).

### 2.2. Molecular Docking and Dynamics Simulation

Bioactive constituents of *Astragalus membranaceus* and their putative targets were retrieved from the TCMSP database [21] (oral bioavailability ≥ 30%, drug-likeness ≥ 0.18). UM-associated targets were collected from the GeneCards database. The intersection targets were subjected to GO function and KEGG pathway enrichment analyses. Molecular docking of ISL, Que and Meth with Gαq, MEK and YAP was performed using AutoDock Vina (v1.5.6) [22,23]. A binding energy of <−7.0 kcal·mol^−1^ was set as the threshold for strong binding. MD simulations were conducted using GROMACS software (v2022) under the CHARMM36 force field with the TIP3P water model. The system was equilibrated at 310 K and 1 bar, with trajectories saved every 2 ps over 100 ns [24,25].

### 2.3. CCK-8 Assay

Cells were seeded into 96-well plates (5 × 10^3^ cells/well) and incubated at 37 °C for 24 h. Following adherence, cells were treated with various concentrations of single agents or combinations for 24, 48 or 72 h in vitro. CCK-8 working solution (100 μL/well; WILBER, cat. no. HC0854) was added and incubated for 2 h. Absorbance at 450 nm was measured using a microplate reader. The CCK-8 assay measures mitochondrial metabolic activity and was employed to generate dose–response matrices required for ZIP synergy quantification.

### 2.4. Synergy Analysis

Two-drug and three-drug combination experiments were designed based on single-agent IC_50_ values determined by CCK-8 assay following 48 h of drug exposure. Synergy was quantified using the ZIP (Zero Interaction Potency) model with SynergyFinder software (https://synergyfinder.fimm.fi (accessed on 3 August 2025)). The ZIP model was selected because it assumes Zero Interaction Potency at all effect levels, providing a robust statistical framework for quantifying combination effects across complete dose–response matrices without requiring parallel dose–response curves—a feature particularly advantageous for evaluating multi-component botanical formulations with non-overlapping mechanisms. A ZIP score >0 indicated synergy, while >10 indicated strong synergy [26,27,28].

Dose–response matrices were constructed using five concentrations per drug, selected above and below the respective IC_50_. In the first stage, all pairwise combinations were screened across complete matrices. In the second stage, the concentration windows yielding the highest synergy scores were used for triple-combination optimization. The final optimal concentrations (92.1: ISL 40 μM + Que 40 μM + Meth 50 μM; C918: ISL 25 μM + Que 40 μM + Meth 60 μM) were selected as the combination achieving the highest ZIP synergy score while exhibiting no significant cytotoxicity against normal ARPE cells compared to the control group. Single-agent and dual-combination controls were tested at equivalent total concentrations for comparative analysis.

### 2.5. Morphological Observation

Cells were plated in 6-well plates at 8 × 10^4^ cells per well. Upon reaching 60–70% confluence, cells were treated with IQM at the optimal synergistic concentrations (92.1: ISL 40 μM + Que 40 μM + Meth 50 μM; C918: ISL 25 μM + Que 40 μM + Meth 60 μM) for 48 h in vitro. Single-agent and dual-combination controls were included at equivalent concentrations. Morphological changes (cell shrinkage, structural disruption or detachment) were observed and photographed under an inverted optical microscope (10× magnification, Olympus, Tokyo, Japan).

### 2.6. Colony Formation Assay

Cells were seeded in 6-well plates at 1 × 10^3^ cells per well. After adherence, cells were exposed to IQM at the optimal synergistic concentrations (92.1: ISL 40 μM + Que 40 μM + Meth 50 μM; C918: ISL 25 μM + Que 40 μM + Meth 60 μM) and cultured for 10 days with medium renewal every 3 days. Single-agent and dual-combination controls were included at equivalent concentrations. Colonies were fixed with 70% ethanol, stained with 1% crystal violet and counted (diameter > 50 μm).

### 2.7. Flow Cytometric Analysis

For cell cycle analysis, cells were treated with IQM (92.1: ISL 40 μM + Que 40 μM + Meth 50 μM; C918: ISL 25 μM + Que 40 μM + Meth 60 μM) for 48 h in vitro, then harvested, fixed in 70% ethanol and stained with propidium iodide PI/RNase A (Liankebio, Hangzhou, China, cat. no. 70-CCS012) for 30 min. For apoptosis detection, cells were treated with the same IQM concentrations for 48 h in vitro, then stained with Annexin V-FITC/PI (Liankebio, cat. no. 70-AP101-100) for 15 min in the dark. Compensation was applied using single-stained compensation controls (Annexin V-FITC only and PI only) to correct for spectral overlap between FITC and PI channels.

### 2.8. Wound Healing Assay

When cells reached 90–100% confluence, a linear scratch was created using a sterile 200 μL pipette tip. After washing with PBS to remove detached cells, serum-free medium containing IQM (92.1: ISL 40 μM + Que 40 μM + Meth 50 μM; C918: ISL 25μM+ Que 40 μM + Meth 60μM) was added. Images were captured at 0 and 24 h using an inverted microscope. Migration rate was calculated as [(width at 0 h–width at 24 h)/width at 0 h] × 100%.

### 2.9. Mitochondrial Membrane Potential Detection

Following 48 h in vitro treatment with IQM (92.1: ISL 40 μM + Que 40 μM + Meth 50 μM; C918: ISL 25 μM + Que 40 μM + Meth 60 μM), cells were incubated with JC-10 (AAT, Pleasanton, CA, USA, cat. no. 22204) working solution at 37 °C for 20 min. After PBS washing, mitochondrial membrane potential was visualized using a laser confocal microscope (Olympus, Japan). Red fluorescence indicated normal membrane potential, whereas green fluorescence indicated depolarization.

### 2.10. Transmission Electron Microscopy (TEM)

Cells were treated with IQM (92.1: ISL 40 μM + Que 40 μM + Meth 50 μM; C918: ISL 25 μM + Que 40 μM + Meth 60 μM) for 48 h in vitro, then fixed with 2.5% glutaraldehyde at 4 °C for 4 h, post-fixed with 1% osmium tetroxide, dehydrated through a graded ethanol series and embedded in Epon 812 resin. Ultrathin sections (70 nm) were cut, stained with uranyl acetate and lead citrate, and examined by TEM to assess mitochondrial ultrastructure.

### 2.11. Western Blot Analysis

Cells were treated with IQM (92.1: ISL 40 μM + Que 40 μM + Meth 50 μM; C918: ISL 25 μM + Que 40 μM + Meth 60 μM) for 48 h in vitro, then lysed in RIPA buffer containing PMSF and phosphatase inhibitors on ice for 30 min. Lysates were centrifuged at 12,000× *g* at 4 °C for 15 min. Protein concentration was determined using a BCA assay. Equal amounts of protein (30 μg) were separated by SDS-PAGE and transferred to PVDF membranes. Following blocking with 5% non-fat milk for 1 h, primary antibodies were diluted according to the manufacturer’s instructions (Table 1) at 4 °C overnight. After washing with TBST, HRP-conjugated secondary antibodies (Servicebio, Wuhan, China, cat. no. GB23303) were applied for 1 h at room temperature. Protein bands were visualized using ECL reagent (Meilunbio, Dalian, China, cat. no. MA0186-1) and quantified using ImageJ software (v2.0.0). Band intensities were measured as integrated density values with background subtraction. The relative expression of each target protein was normalized to the corresponding β-actin loading control and expressed as fold change relative to the control group. Data are presented as mean ± standard deviation (SD) from at least three independent experiments.

### 2.12. Establishment of UM Mouse Model and Treatment

Male BALB/c nude mice (age, 5–6 weeks; weight, 18–20 g) were obtained from Cavens Laboratory Animal Co. and housed under specific pathogen-free conditions at 22 ± 2 °C with a 12 h light/dark cycle. A suspension of 2 × 10^6^ C918 cells in 200 μL PBS was subcutaneously inoculated into the right flank. Tumor-bearing mice were randomly allocated into three groups (n = 6 per group, total 18 mice), using a random number table, when tumors reached approximately 5 mm in diameter. Sample size was determined based on previous xenograft studies. Cage location was randomized to minimize potential confounders. Blinding was not performed due to the visible differences in drug solutions. No exclusion criteria were established and no animals were excluded from the analysis. The primary outcome measure was tumor volume. Groups were as follows: control (normal saline, intraperitoneal injection every other day); IQM (15 mg/kg ISL + 24 mg/kg Que + 36 mg/kg Meth, intraperitoneal every other day); and Trametinib (1 mg/kg, intraperitoneal twice weekly). Treatments lasted for 14 days. The intraperitoneal dosing regimen (ISL 15 mg/kg + Que 24 mg/kg + Meth 36 mg/kg, in a 5:8:12 ratio, every other day) was selected by maintaining the relative proportion of the in vitro optimal synergistic concentrations (C918 cells: ISL 25 μM + Que 40 μM + Meth 60 μM; the proportion is 5:8:12), with individual doses constrained to literature-reported effective ranges and determined through preliminary tolerability assessment [29,30,31,32]. The intraperitoneal route and every-other-day schedule were chosen to bypass the poor oral bioavailability and rapid metabolism characteristics of these flavonoids [33,34,35]. Tumor dimensions and body weight were measured every 2 days. Tumor volume (mm^3^) was calculated as follows: Tumor volume = (length × width^2^)/2. At the endpoint, mice were euthanized via CO_2_ inhalation with a flow rate of 60% chamber volume/min, and death was verified by respiratory cessation. Tumors were excised and weighed. Humane endpoints were defined as tumor volume >2000 mm^3^ or body weight loss >20%. Mice were monitored daily for signs of distress. All animal experiments were approved by the Experimental Animal Ethics Committee of The First Hospital of Lanzhou University (approval no. LDYYLL2026-92, Lanzhou, China). All procedures were carried out under ARRIVE guidelines (https://arriveguidelines.org (accessed on 5 December 2025)).

### 2.13. Serum Biochemical Analysis

At the experimental endpoint, blood was collected via retro-orbital bleeding from a randomly selected subset of mice (n = 3 per group). Serum was separated and analyzed using an automated biochemical analyzer to determine liver function markers (ALT, AST) and renal function markers (BUN, CREA).

### 2.14. Immunohistochemistry (IHC) and Immunofluorescence (IF)

Tumor tissues were randomly sampled from three mice per group (n = 3), fixed in 4% paraformaldehyde, paraffin-embedded and sectioned (4 μm). Sections were dewaxed, rehydrated, subjected to antigen retrieval, blocked with 3% H2O2 and 5% BSA, and incubated with primary antibodies at 4 °C overnight. For IHC, sections were incubated with HRP-conjugated secondary antibody, developed with DAB and counterstained with hematoxylin. For IF, sections were incubated with FITC-conjugated secondary antibody in the dark for 1 h, counterstained with DAPI and mounted with anti-fade medium for confocal microscopy observation.

### 2.15. Statistical Analysis

All data are expressed as the mean ± standard deviation. Comparisons between two groups were performed using Student’s *t*-test, while those among multiple groups were analyzed by one-way ANOVA followed by Tukey’s post hoc test. *p* < 0.05 was considered to indicate a statistically significant difference. All analyses were performed using SPSS 22.0 software. Prior to parametric analyses, normality was assessed using the Shapiro–Wilk test and homogeneity of variance was assessed using Levene’s test. When normality was violated, data were analyzed using non-parametric alternatives (e.g., Kruskal–Wallis test).

## 3. Results

### 3.1. Molecular Validation of ISL, Que and Meth Binding to Gαq, MEK and YAP

**Figure 1:**
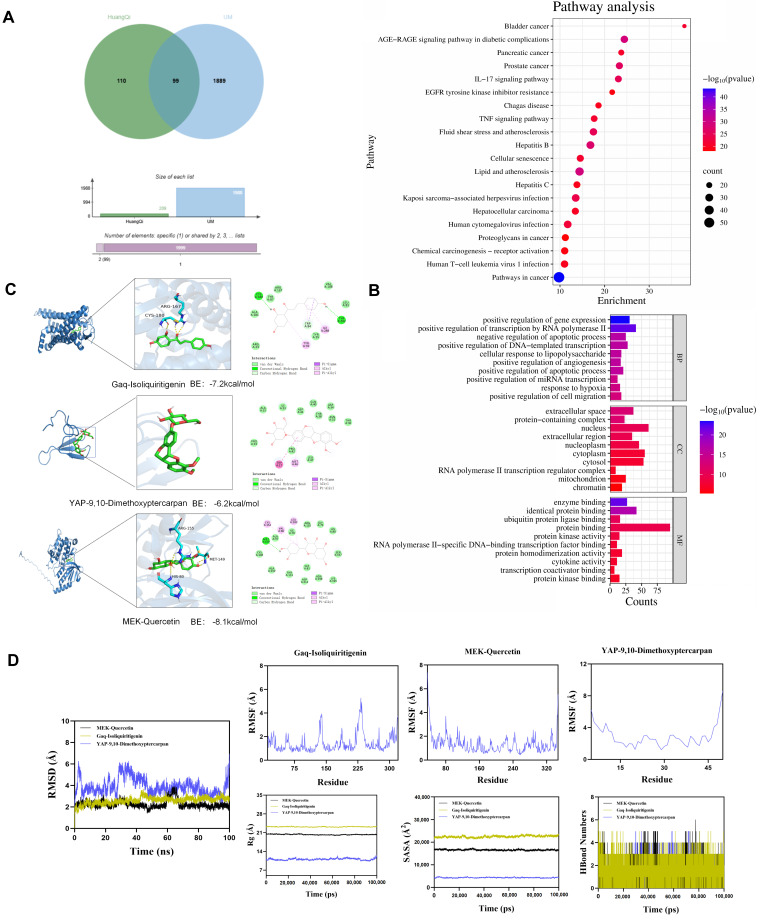
Molecular validation of ISL, Que, and Meth binding to Gαq, MEK, YAP. Network pharmacology analysis identified intersection targets between *Astragalus membranaceus* constituents and UM. (**A**) Venn diagram of overlapping targets. (**B**) GO and KEGG enrichment analysis. (**C**) Molecular docking patterns and binding energies (kcal·mol^−1^) of ISL-Gαq, MEK-Que and Meth-YAP complexes. (**D**) MD simulation trajectories (RMSD, Rg, SASA, RMSF and hydrogen bond analysis).

Network pharmacology analysis identified intersection targets between *Astragalus membranaceus* constituents and UM (Figure 1A), and GO/KEGG enrichment analysis is shown in Figure 1B. Molecular docking analysis revealed favorable binding affinities between ISL and Gαq, and Que and MEK, as well as Meth and YAP (binding energies, <−7.0 kcal·mol^−1^; Figure 1C). MD simulation trajectories demonstrated that all three drug–target complexes maintained stable conformations over 100 ns (RMSD, <3 Å; Figure 1D), confirming reliable binding modes.

### 3.2. Effects of Single Agents on Proliferation and Target Proteins in UM Cells

**Figure 2:**
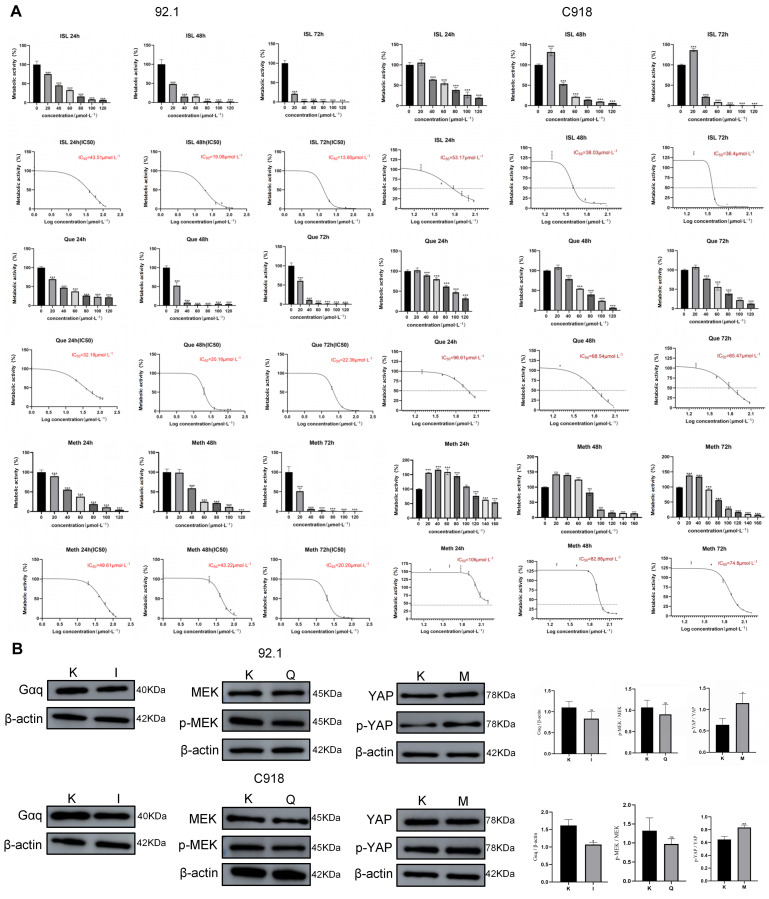
Effects of ISL, Que and Meth on proliferation and target proteins in UM cells. (**A**) Metabolic activity of 92.1 and C918 cells treated with ISL, Que or Meth for 24, 48 and 72 h, as determined by CCK-8 assay. (**B**) Western blot analysis of Gαq, MEK, p-MEK (activated form), YAP and p-YAP (inactive form, cytoplasmic retention) protein levels. n = 3. ns, not significant; * *p* < 0.05, ** *p* < 0.01, *** *p* < 0.001 vs. control.

Treatment with ISL, Que or Meth significantly reduced the metabolic activity of 92.1 and C918 cells in a dose- and time-dependent manner (Figure 2A). Western blot analysis revealed that ISL showed a tendency to decrease total Gαq protein expression, whereas Que suppressed MEK phosphorylation (p-MEK). Conversely, Meth increased YAP phosphorylation (p-YAP), promoting its cytoplasmic retention and subsequent degradation, thus attenuating YAP-mediated transcriptional activity (Figure 2B). These findings indicated that each compound effectively modulates its intended target in UM cells. The combined effect of the triple combination (IQM) on these targets is presented in the corresponding results (in vitro and in vivo).

### 3.3. Synergistic Interaction and Selectivity of IQM

**Figure 3:**
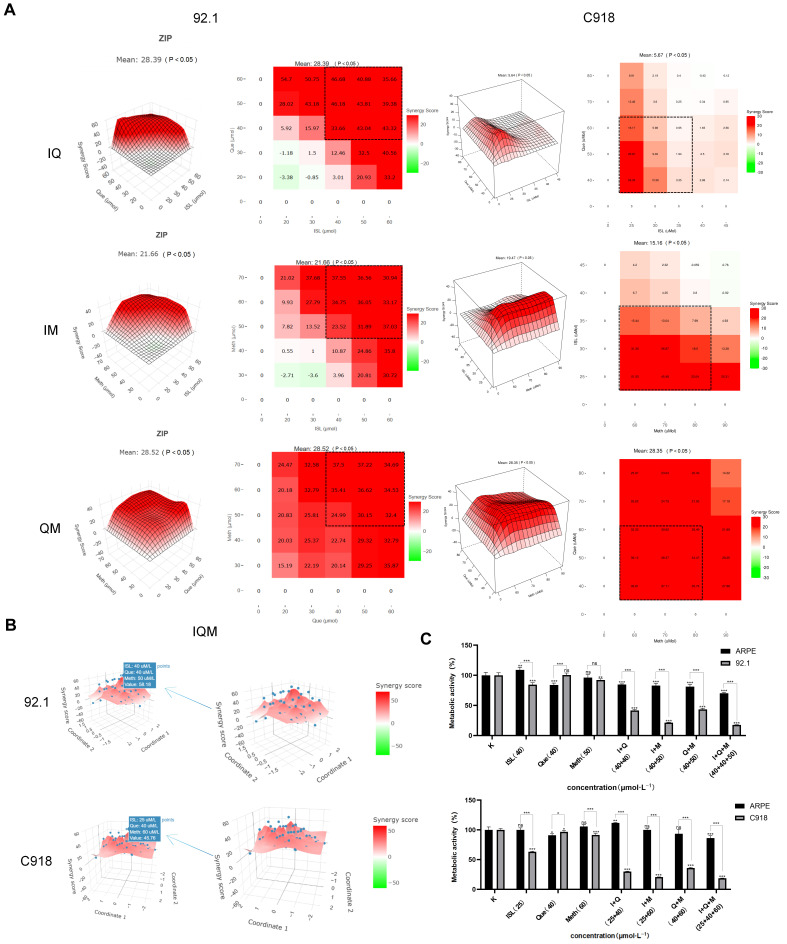
Synergistic interaction of IQM in UM cells based on CCK-8 assays. (**A**) Synergy analysis of pairwise combinations in 92.1 and C918 cells (ZIP scores, 48 h data). The dashed boxes indicate the concentration ranges with higher ZIP synergy scores. (**B**) Synergy scores of triple combination (IQM) at optimal concentrations (92.1: ISL 40 μM + Que 40 μM + Meth 50 μM; C918: ISL 25 μM + Que 40 μM + Meth 60 μM) (ZIP scores, 48 h data). (**C**) Metabolic activity of normal ARPE cells and UM cells treated with triple combination for 48 h in vitro. n = 3. ns, not significant; * *p* < 0.05, ** *p* < 0.01, *** *p* < 0.001 vs. control.

Synergy analysis using the ZIP model revealed that all pairwise combinations (ISL + Que, ISL + Meth, Que + Meth) exhibited strong synergistic effects (ZIP scores, >10; Figure 3A). Notably, the triple combination (IQM) achieved higher synergy scores (58.18 in 92.1 cells and 45.76 in C918 cells) compared with dual combinations (Figure 3B). Additionally, the triple combination exerted minimal cytotoxicity against normal ARPE cells (metabolic activity reduction <10% at the tested concentration and 48 h in vitro time point, indicating favorable tumor selectivity under these assay conditions (Figure 3C)).

### 3.4. IQM Suppresses UM Cell Proliferation, Cell Cycle Progression and Migration

**Figure 4:**
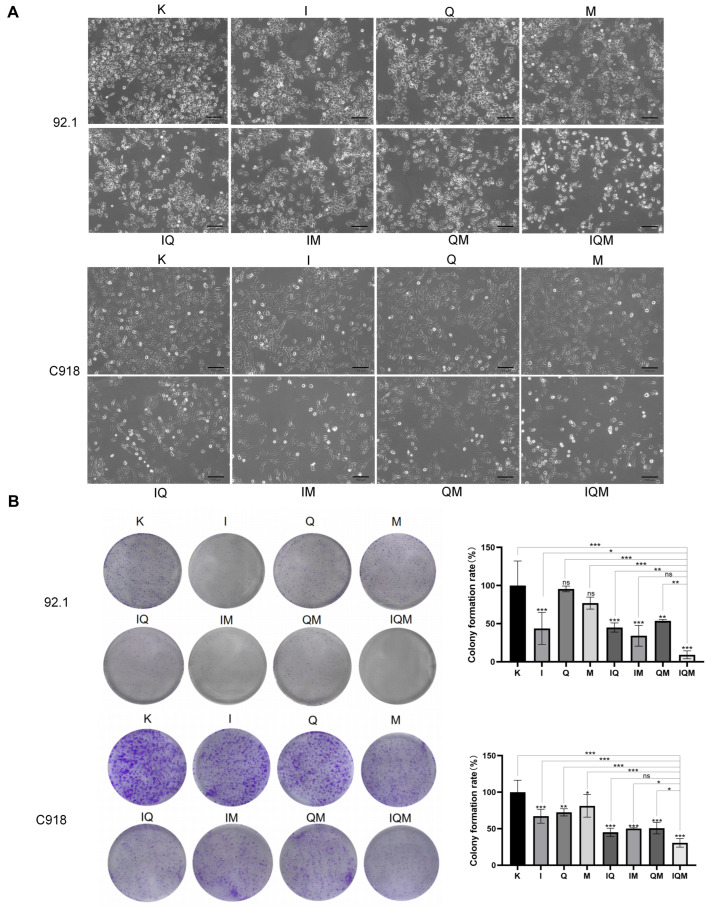
Effect of IQM on UM cell morphology and clonogenic survival. (**A**) Representative images of 92.1 and C918 cell morphology after 48 h in vitro treatment with IQM (magnification, ×10). (**B**) Colony formation assays; representative images and quantification of colonies (>50 μm). IQM concentrations were as specified in Section 2.4. n = 3. ns, not significant; * *p* < 0.05, ** *p* < 0.01, *** *p* < 0.001 vs. control.

**Figure 5:**
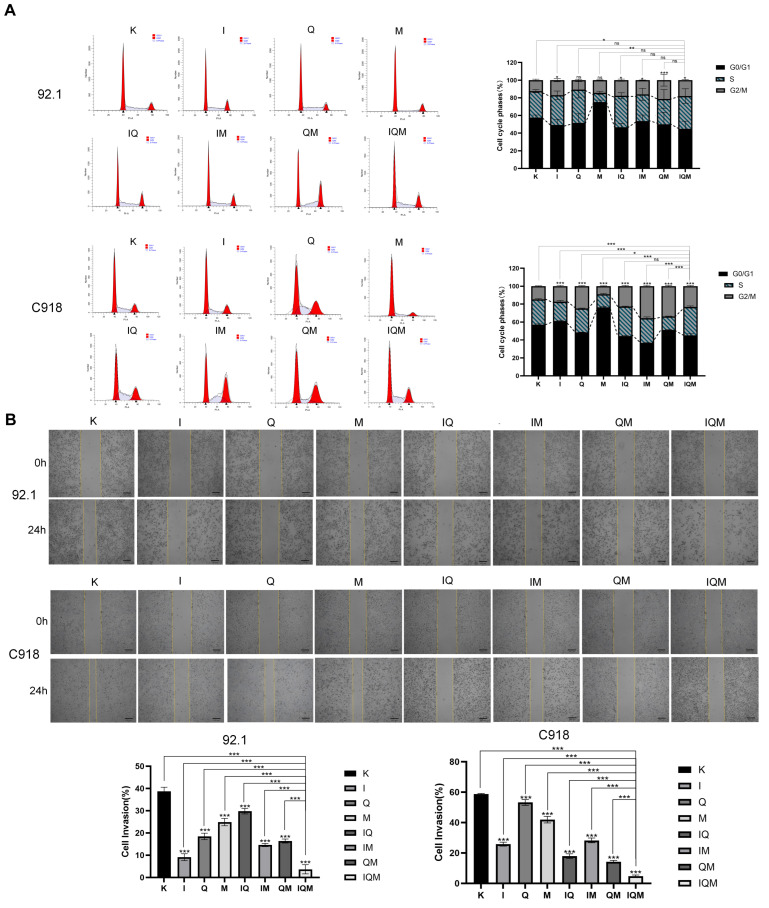
Effect of IQM on cell cycle and migration in UM cells. (**A**) Cell cycle distribution following 48 h in vitro treatment with IQM. (**B**) Wound healing assay at 0 and 24 h and quantitative analysis. IQM concentrations were as specified in Section 2.4. n = 3. ns, not significant; * *p* < 0.05, ** *p* < 0.01, *** *p* < 0.001 vs. control.

Following 48 h in vitro treatment, morphological examination revealed that triple-combination-treated cells exhibited marked shrinkage, blurred boundaries and detachment, characteristic of apoptotic morphology (Figure 4A). Consistent with these observations, colony formation assays demonstrated that the triple combination significantly reduced colony numbers compared with single- or dual-drug groups (Figure 4B). Furthermore, flow cytometric analysis indicated that the triple combination caused a modest increase in G2/M phase population in both cell lines (Figure 5A), although the magnitude of arrest was relatively limited compared with the robust apoptotic induction observed. In addition, wound healing assays revealed that the triple combination markedly suppressed cell migratory capacity (Figure 5B).

### 3.5. IQM Induces Caspase 9-Dependent Apoptosis via the Intrinsic Mitochondrial Pathway

**Figure 6:**
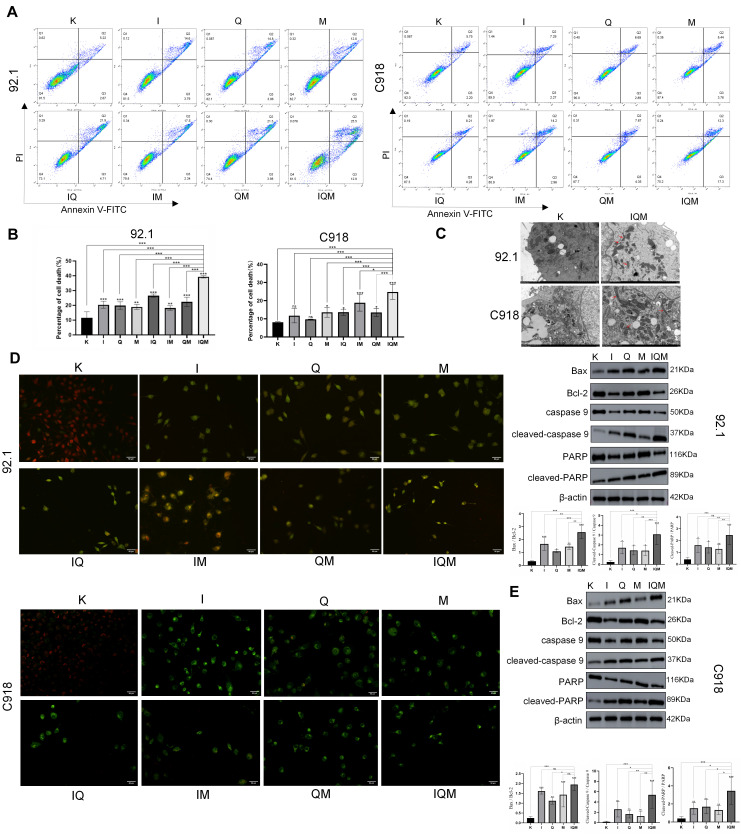
In vitro effects of IQM on caspase 9-dependent apoptosis via the intrinsic mitochondrial pathway. (**A**,**B**) Flow cytometric analysis of apoptotic cells (Annexin V/PI staining) following 48 h in vitro treatment with IQM. (**C**) Transmission electron micrographs of mitochondrial ultrastructure (magnification, ×8000, 48 h in vitro treatment with IQM). Red arrows indicate apoptotic mitochondrial alterations (cristae disruption and matrix condensation). (**D**) JC-10 staining of mitochondrial membrane potential (48 h treatment in vitro with IQM). (**E**) Western blot analysis of Bcl-2, Bax, caspase 9, cleaved-caspase 9, PARP and cleaved-PARP (48 h in vitro treatment with IQM). IQM concentrations were as specified in Section 2.4. n = 3. ns, not significant; * *p* < 0.05, ** *p* < 0.01, *** *p* < 0.001 vs. control.

Flow cytometric analysis revealed that the triple combination significantly increased the total apoptotic rate compared with mono- or dual-therapy groups (Figure 6A,B). JC-10 staining indicated discernible collapsing trends of mitochondrial membrane potential in the triple-combination group, as reflected by the elevated green/red fluorescence ratio (Figure 6D). Consistent with these findings, transmission electron microscopy revealed characteristic apoptotic mitochondrial alterations, including cristae disruption and matrix condensation (Figure 6C). Moreover, Western blot analysis demonstrated that the triple combination significantly upregulated pro-apoptotic Bax and downregulated anti-apoptotic Bcl-2, leading to activation of caspase 9 and cleavage of its substrate PARP (Figure 6E). These results suggested that the triple combination promotes UM cell apoptosis through activation of caspase 9-dependent apoptosis via the intrinsic mitochondrial pathway.

### 3.6. Anti-Tumor Efficacy of IQM in a UM Xenograft Model

**Figure 7:**
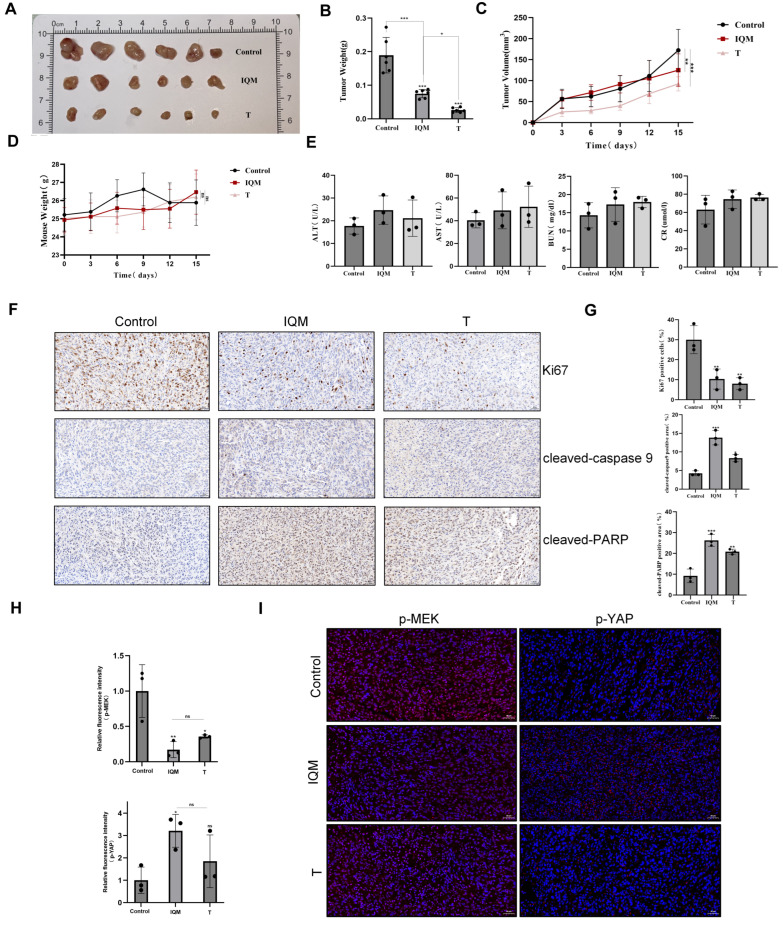
Anti-tumor efficacy of IQM in a UM xenograft model. C918 xenograft mice were treated with control, IQM (15 mg/kg ISL + 24 mg/kg Que + 36 mg/kg Meth, intraperitoneal, every other day) or Trametinib (1 mg/kg, intraperitoneal, twice weekly) for 14 days. (**A**) Representative tumor images and volumes. (**B**) Tumor weights and inhibition rates. (**C**) Tumor growth curves. (**D**) Mouse weight. (**E**) Serum levels of ALT, AST, BUN and CREA. (**F**,**G**) IHC of Ki67, cleaved-caspase 9 and cleaved-PARP. (**H**,**I**) IF of p-MEK and p-YAP (red) with DAPI (blue). n = 6 for (**A**–**D**); n = 3 for (**E**–**H**). ns, not significant; * *p* < 0.05, ** *p* < 0.01, *** *p* < 0.001 vs. control.

In the C918 xenograft model, the triple combination significantly suppressed tumor growth compared with the control group. Notably, the anti-tumor efficacy of IQM was comparable to that of Trametinib, a clinically approved MEK inhibitor (Figure 7A–C). We explicitly note that this represents preliminary proof-of-concept activity in a short-term xenograft study, not a definitive therapeutic endpoint. However, because the xenograft design did not include single-agent or dual-combination arms, these data demonstrate the therapeutic efficacy of the triple regimen but do not prove that the observed effect is synergistic rather than additive in vivo. Notably, no significant differences in body weight or serum biochemical markers (ALT, AST, BUN, CREA) were observed between groups, indicating favorable systemic safety (Figure 7D,E). Consistent with the in vitro findings, immunohistochemical analysis revealed reduced Ki67 proliferation index and elevated cleaved-caspase 9 and cleaved-PARP expression in tumors from the triple-combination group (Figure 7F,G). Furthermore, immunofluorescence staining demonstrated decreased p-MEK and increased p-YAP fluorescence intensity in the treatment group (Figure 7H,I), confirming effective inhibition of MEK phosphorylation and promotion of YAP cytoplasmic retention in vivo. Collectively, these results demonstrated that IQM effectively suppresses tumor progression in vivo via suppression of proliferation and induction of apoptosis.

## 4. Discussion

The present study provides the first demonstration that ISL-Que-Meth synergistically suppresses UM growth in vitro in association with coordinated modulation of Gαq/MEK/YAP axis components and activation of caspase 9-dependent apoptosis via the intrinsic mitochondrial pathway. This multi-targeted strategy addresses a fundamental therapeutic vulnerability of UM: single-node inhibition frequently triggers compensatory feedback activation, whereas simultaneous blockade of upstream signal initiation (Gαq), pathway amplification (MEK) and transcriptional execution (YAP) minimizes escape routes. The superior efficacy of the triple combination over monotherapy reflects both comprehensive pathway coverage and the non-overlapping target specificity of its botanical constituents [36,37,38,39,40,41].

Synergy was quantified using the ZIP model, which was selected because it provides a robust statistical framework for quantifying combination effects across full dose–response matrices without assuming parallel dose–response curves, making it particularly suitable for evaluating multi-component botanical formulations with distinct target specificities (Figure 3B). We emphasize that CCK-8 screening enabled high-throughput matrix generation, while clonogenic assays independently validated long-term tumor cell eradication as the definitive biological endpoint. The efficacy observed in both *GNAQ*-mutant (92.1) and wild-type (C918) cell lines indicates that this strategy targets Gαq signaling activity rather than being limited to oncogenic mutant subsets, thereby extending its potential applicability to a broader UM patient population.

While the convergence of mitochondrial membrane potential collapse, cristae disruption, Bax/Bcl-2 imbalance, caspase 9 activation and PARP cleavage strongly supports intrinsic apoptosis as the dominant cytotoxic mechanism, the modest G2/M phase accumulation appears secondary to apoptotic execution rather than a primary growth-arrest mechanism. We acknowledge that direct biophysical validation of compound–target binding (e.g., cellular thermal shift assay or surface plasmon resonance), rescue experiments with constitutively active mutants, and formal exclusion of extrinsic apoptosis and alternative programmed cell death modalities (necroptosis, pyroptosis, ferroptosis) were not performed and represent essential priorities for follow-up studies.

Importantly, the in vivo xenograft results reflect integrated pharmacodynamic responses under physiological drug clearance and tumor microenvironment conditions, fundamentally differing from the constant-concentration 48 h in vitro exposure paradigm. Consequently, in vitro synergy data should be interpreted as proof-of-concept for combination potential, whereas xenograft results represent preliminary efficacy under pharmacokinetically complex conditions. The C918 xenograft model demonstrated significant tumor suppression and favorable systemic safety following 14 days of treatment. Nevertheless, the approximately 50% growth inhibition observed, while statistically significant compared with the control group, is biologically modest and would be insufficient for definitive tumor eradication in a rapidly proliferating malignancy such as UM. This observation is explicitly framed as preliminary proof-of-concept evidence to justify further dose-escalation and schedule-optimization studies, rather than as a claim of clinically meaningful efficacy. Moreover, the absence of single-agent and dual-combination arms precludes definitive claims of in vivo synergy, and pharmacodynamic assessment was limited to semi-quantitative IHC/IF without quantitative tumor lysate Western blot or plasma/tissue compound measurements. The intraperitoneal dosing regimen (ISL 15 mg/kg + Que 24 mg/kg + Meth 36 mg/kg, every other day) was selected to bypass the poor oral bioavailability and rapid metabolism characteristic of these flavonoids, but comprehensive pharmacokinetic/pharmacodynamic (PK/PD) profiling, dose–response matrix optimization, and advanced formulation development will be essential prerequisites for clinical translation.

The use of immunodeficient nude mice, which lack adaptive immunity, represents a non-physiological preclinical model with limited predictive value for clinical outcomes. This limitation precluded evaluation of immune-related anti-tumor effects, a relevant consideration given the established immunomodulatory properties of *Astragalus membranaceus* constituents. Future studies in immunocompetent syngeneic models and exploration of combinations with immune checkpoint blockade are warranted to determine whether IQM-induced apoptosis enhances anti-tumor immunity.

While the present study establishes preclinical proof-of-concept for IQM through integrated target prediction, in vitro synergy validation, and preliminary in vivo efficacy assessment, we acknowledge that the observed Gαq/MEK/YAP axis modulation and apoptotic induction represent correlational rather than causally validated observations. Future studies will therefore be required to formally establish mechanistic causality through genetic rescue experiments and pathway-specific pharmacological perturbation, to elucidate the pharmacokinetic–pharmacodynamic relationship bridging in vitro exposure and in vivo drug behavior, and to expand the safety profile through comprehensive multi-dose and chronic toxicity assessments. These directions represent the logical continuation of our research program as we advance IQM from a candidate combination toward a mechanistically validated therapeutic strategy.

In summary, IQM establishes a preclinical rationale for multi-targeted natural product therapy against UM. The multi-layered evidence—spanning computational target prediction, in vitro synergy quantification with clonogenic validation, mechanistic pathway analysis, and preliminary in vivo proof-of-concept—provides a foundation for subsequent validation of target engagement, rigorous PK/PD correlation, and formulation optimization. Addressing these priorities through rigorous biophysical binding confirmation, comprehensive drug exposure–efficacy relationship characterization, and clinically viable delivery system development will be essential to advance IQM from preclinical proof-of-concept toward potential clinical evaluation against this genetically complex and therapeutically challenging malignancy.

## 5. Conclusions

The present study demonstrates that the triple combination of ISL, Que and Meth synergistically suppresses UM growth in vitro in association with coordinated modulation of the Gαq/MEK/YAP axis and activation of caspase 9-dependent apoptosis via the intrinsic mitochondrial pathway. Significant anti-tumor efficacy and favorable systemic safety were also observed in the C918 xenograft model; however, definitive proof of in vivo synergy awaits future studies incorporating single-agent and dual-combination arms. It is critical to distinguish between the in vitro findings—where cells are exposed to constant drug concentrations for 48 h—and the in vivo xenograft results, which reflect integrated pharmacodynamic responses under physiological drug clearance. Accordingly, the in vitro synergy data provide proof-of-concept for combination potential, while the in vivo data demonstrate preliminary efficacy under clinically relevant but pharmacokinetically complex conditions. Clinical translation would additionally require comprehensive pharmacokinetic/pharmacodynamic optimization and advanced formulation development to address the bioavailability limitations inherent to these natural product constituents.

## Figures and Tables

**Table 1:** Antibodies for Western blotting.

Name	Supplier	Cat. No.
Gαq	Proteintech (Rosemont, IL, USA)	13927-1-AP
Mek	Servicebio	GB15600
p-Mek	Servicebio	GB115603
p-YAP	Servicebio	GB114060
YAP	Servicebio	GB153975
Bax	Servicebio	GB15690
Bcl-2	Servicebio	GB154380
Caspase9	Servicebio	GB115730
Cleaved-Caspase9	CST (Danvers, MA, USA)	#9509
Cleaved-PARP	Servicebio	GB151503
PARP	ABCAM (Cambridge, UK)	AB32064

## Data Availability

The original contributions presented in this study are included in the article/Supplementary Material. Further inquiries can be directed to the corresponding author.

## Supplementary Materials

The following supporting information can be downloaded at: https://www.mdpi.com/article/10.3390/biomedicines14071596/s1, Data S1: C918 STR Report; Data S2: Cellosaurus database entry for 92.1 cell line (CVCL_8607).
