# Supplementary material for: Combination of Three Herbal Components (ISL, Que, Meth) Suppresses Uveal Melanoma Growth via Gαq/MEK/YAP Axis Modulation and Apoptosis
Source: Biomedicines. 2026 Jul 16;14(7):1596. doi: 10.3390/biomedicines14071596 (PMC13406481; doi:10.3390/biomedicines14071596)
Supplement: Supplementary file 1 [file biomedicines-14-01596-s001.zip › Supplementary Data S1-C918 STR Report.pdf]

C918 Cell STR Authentication Report

Note: This is an English translation of the original STR report for C918 cells (catalog no. PC-H2025103038), issued by Procell Life Science & Technology Co., Ltd.

I. Sample Processing and Testing Methods

An appropriate amount of C918 cells (catalog no. PC-H2025103038, 1×10^6) was used for DNA extraction with the TIANamp Genomic DNA Kit. A total of 20 STR loci and one sex-determination locus were amplified using the Microreader™ 21 ID System. PCR products were detected with the GenReader_7010 genetic analyzer, and the results were analyzed using GeneMapper Software 6 (Applied Biosystems) and compared against the ExPASy database.

II. Testing Results

Negative and positive controls in the experiment were both correct.

The STR loci and Amelogenin locus genotyping results for the C918 cell line are shown in the attached table, and the electropherograms are shown in the attached figures.

III. Analysis Notes

The genomic DNA of the C918 cell line was successfully amplified, and the genotyping results were of good quality.

IV. Testing Conclusions

1. 1. STR profiling of the C918 cell line DNA showed **no human cell cross-contamination** in the cell line.
2. 2. The DNA profile of this cell line matched a cell line in the database with **97.78% similarity**. The matched cell line is designated **C918**.

Attached Table 1: Genotyping Results of STR Loci and Amelogenin Locus for C918 Cell Line

**STR Loci**	**Sample Name: PC-H2025103038**	**Database Name: C918**
Amelogenin	X	X
CSF1PO	10,11	10,11
D2S1338	24,25	24,25
D3S1358	16	16

D5S818	11,12	11,12
D7S820	12	12
D8S1179	13	13
D13S317	11	11
D16S539	8,12	8,12
D18S51	15	15
D19S433	13,14	13,14
D21S11	30	30
FGA	21	20,21
Penta D	9,13	—
Penta E	7,13	—
TH01	8,9.3	8,9.3
TPOX	11	11
vWA	16,17	16,17
D6S1043	11,12	—
D12S391	17,22	—
D2S441	11,14	—

ExPASy database match: 97.78%, matching loci: 15 (https://www.cellosaurus.org/index.html)

Notes:

1. According to the STR authentication standards established by the International Cell Line Authentication Committee (ICLAC), a match of **≥80%** indicates that the cell lines are related (derived from a common ancestral cell); a match between **55% and 80%** requires further verification of relatedness; and a match of **<55%** indicates that the two cell lines are not related.
2. Valid peaks in the electropherogram represent authentic PCR products; minor peaks and non-specific bands are ignored in the calculation.
3. STR data comparison results are based on the ExPASy database by default. Data sources include ATCC, DSMZ, JCRB, and other cell banks, as well as literature and documented records. Database portal: https://www.cellosaurus.org/index.html.

Attached Figure S1: Genotyping results of STR loci and Amelogenin locus for C918 cells (catalog no. PC-H2025103038).

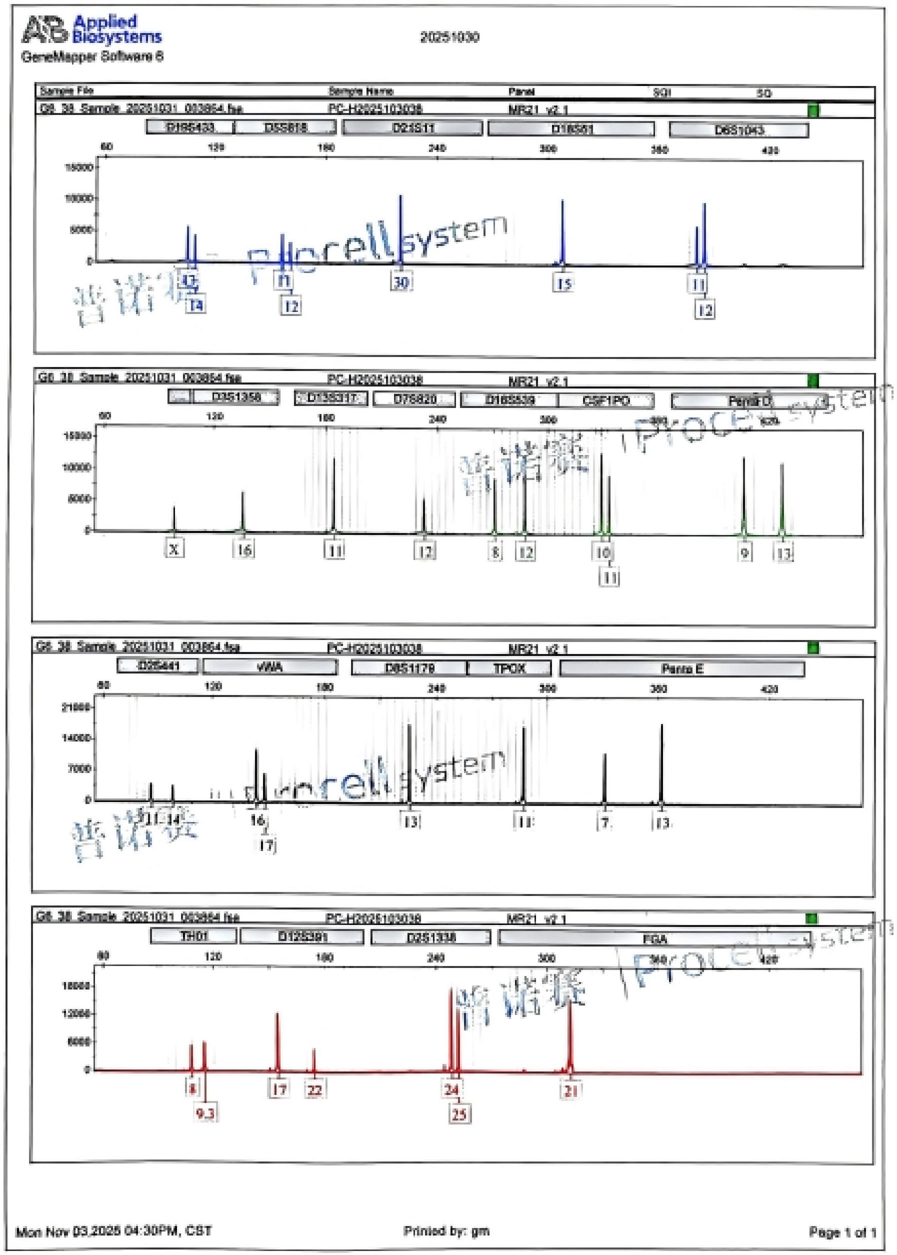

Procell Life Science & Technology Co., Ltd.
Website: www.procell.com.cn
Tel: 400-999-2100
Email: techsupport@procell.com.cn
Address: Building C4, Bio-Pharmaceutical Industrial Park, No. 858 Gaoxin Avenue, Wuhan, Hubei, China
