# Supplementary material for: Combination of Three Herbal Components (ISL, Que, Meth) Suppresses Uveal Melanoma Growth via Gαq/MEK/YAP Axis Modulation and Apoptosis
Source: Biomedicines. 2026 Jul 16;14(7):1596. doi: 10.3390/biomedicines14071596 (PMC13406481; doi:10.3390/biomedicines14071596)
Supplement: Supplementary file 1 [file biomedicines-14-01596-s001.zip › Supplementary Data S2-Cellosaurus database entry for 92.1 cell line (CVCL_8607).pdf]

Cellosaurus 92-1 [Human uveal melanoma] (CVCL_8607)

[Text][XML][JSON]

Cell line name	92-1 [Human uveal melanoma]
Synonyms	92_1; 92.1; 921
Accession	CVCL_8607
Resource Identification Initiative	To cite this cell line use: 92-1 [Human uveal melanoma] (RRID:CVCL_8607)
Comments	Part of: Cancer Dependency Map project (DepMap) (includes Cancer Cell Line Encyclopedia - CCLE).<br>Characteristics: Exhibits a mesenchymal phenotype (PubMed=38601020).<br>Doubling time: 58 hours (PubMed=7622289); 38 hours (PubMed=24994677).<br>Omics: Proteomics.<br>Omics: Proteomics; Quantitative.<br>Omics: Transcriptomics; Microarray.<br>Omics: Transcriptomics; RNAseq.<br>Derived from site: In situ; Eye, uvea; UBERON=UBERON_0001768.
Sequence variations	• Mutation; HGNC; HGNC:3250; EIF1AX; Simple; p.Gly6Asp (c.17G>A); Zygosity=Unspecified (PubMed=39042403).<br>• Mutation; HGNC; HGNC:4390; GNAQ; Simple; p.Gln209Leu (c.626A>T); ClinVar=VCV000375955; Zygosity=Heterozygous (PubMed=22236444; PubMed=22383533; PubMed=23851445; PubMed=24994677; PubMed=28018010).
HLA typing	Source: PubMed=28018010<br>Class I<br>HLA-A: A*02:01,03:01<br>HLA-B: B*44:02,51:01<br>HLA-C: C*05:01,14:02
Disease	Uveal melanoma (NCIt: C7712)<br>Uveal melanoma (ORDO: Orphanet_39044)
Species of origin	Homo sapiens (Human) (NCBI Taxonomy: 9606)
Hierarchy	Children:<br>CVCL_C4PP (92.1-A); CVCL_C4PQ (92.1-B)
Originate from same individual	CVCL_C300 ! 92-2 [Human uveal melanoma]
Sex of cell	Female
Age at sampling	76Y
Category	Cancer cell line

Category	Cancer cell line

STR profile

Source(s): DepMap=ACH-001441; ECACC=13012458; ESTDAB=ESTDAB-127; PubMed=22236444; PubMed=24994677; PubMed=28018010

Markers:

Amelogenin	X
CSF1PO	10,11
D3S1358	14,15
D5S818	9,11 (DepMap=ACH-001441; ECACC=13012458; PubMed=22236444; PubMed=24994677)<br>12,13 (ESTDAB=ESTDAB-127)
D7S820	10,11 (DepMap=ACH-001441; ECACC=13012458; PubMed=22236444; PubMed=24994677)<br>11 (ESTDAB=ESTDAB-127)
D8S1179	15
D13S317	10,11 (ESTDAB=ESTDAB-127; PubMed=24994677)<br>11,12 (DepMap=ACH-001441; ECACC=13012458; PubMed=22236444)
D16S539	11,12 (PubMed=22236444; PubMed=24994677)<br>12 (DepMap=ACH-001441; ECACC=13012458)
D18S51	12,13
D21S11	30
FGA	21,23
Penta D	9,11
Penta E	12,14
TH01	9,9.3
TPOX	8,9
vWA	16

Run an STR similarity search on this cell line

Cell line collections (Providers)	ECACC; 13012458<br>ICLC; HTL12001
Cell line databases/resources	CLDB; cl7221<br>cancercelllines; CVCL_8607<br>DepMap; ACH-001441<br>ESTDAB; ESTDAB-127
Encyclopedic resources	Wikidata; Q54605663
Experimental variables resources	EFO; EFO_0022503
Gene expression databases	GEO; GSM276761
Polymorphism and mutation databases	Cosmic; 848328<br>Cosmic; 899893<br>Cosmic; 916137<br>Cosmic; 986619<br>Cosmic; 1320371<br>Cosmic; 1628394<br>Cosmic; 1669117<br>Cosmic; 2036703<br>Cosmic; 2163829
Proteomic databases	PRIDE; PXD032215<br>PRIDE; PXD051055
**Entry history**	
Entry creation	04-Apr-2012
Last entry update	14-Aug-2025
Version number	39
